# Tethering of Epidermal Growth Factor (EGF) to Beta Tricalcium Phosphate (βTCP) via Fusion to a High Affinity, Multimeric βTCP-Binding Peptide: Effects on Human Multipotent Stromal Cells/Connective Tissue Progenitors

**DOI:** 10.1371/journal.pone.0129600

**Published:** 2015-06-29

**Authors:** Luis M. Alvarez, Jaime J. Rivera, Linda Stockdale, Sunil Saini, Richard T. Lee, Linda G. Griffith

**Affiliations:** 1 Department of Biological Engineering, Massachusetts Institute of Technology, Cambridge, Massachusetts, United States of America; 2 Integra Life Sciences, Plainsboro, New Jersey, United States of America; 3 Department of Stem Cell and Regenerative Biology, Harvard University, Cambridge, Massachusetts, United States of America; 4 Department of Chemistry & Life Science, United States Military Academy, West Point, New York, United States of America; 5 Cancer and Developmental Biology Laboratory, National Cancer Institute, Frederick, Maryland, United States of America; Institute for Frontier Medical Sciences, Kyoto University, JAPAN

## Abstract

Transplantation of freshly-aspirated autologous bone marrow, together with a scaffold, is a promising clinical alternative to harvest and transplantation of autologous bone for treatment of large defects. However, survival proliferation, and osteogenic differentiation of the marrow-resident stem and progenitor cells with osteogenic potential can be limited in large defects by the inflammatory microenvironment. Previous studies using EGF tethered to synthetic polymer substrates have demonstrated that surface-tethered EGF can protect human bone marrow-derived osteogenic stem and progenitor cells from pro-death inflammatory cues and enhance their proliferation without detriment to subsequent osteogenic differentiation. The objective of this study was to identify a facile means of tethering EGF to clinically-relevant βTCP scaffolds and to demonstrate the bioactivity of EGF tethered to βTCP using stimulation of the proliferative response of human bone-marrow derived mesenchymal stem cells (hBMSC) as a phenotypic metric. We used a phage display library and panned against βTCP and composites of βTCP with a degradable polyester biomaterial, together with orthogonal blocking schemes, to identify a 12-amino acid consensus binding peptide sequence, LLADTTHHRPWT, with high affinity for βTCP. When a single copy of this βTCP-binding peptide sequence was fused to EGF via a flexible peptide tether domain and expressed recombinantly in *E*. *coli* together with a maltose-binding domain to aid purification, the resulting fusion protein exhibited modest affinity for βTCP. However, a fusion protein containing a linear concatamer containing 10 repeats of the binding motif the resulting fusion protein showed high affinity stable binding to βTCP, with only 25% of the protein released after 7 days at 37^o^C. The fusion protein was bioactive, as assessed by its abilities to activate kinase signaling pathways downstream of the EGF receptor when presented in soluble form, and to enhance the proliferation of hBMSC when presented in tethered form on commercial βTCP bone regeneration scaffolds.

## Introduction

Bone grafting procedures in the USA top the half-million mark annually and 2.2 million worldwide [1,2]. They represent an approximate 1.5 billion dollar industry in the USA alone [1,2]. These procedures are a requirement for healing of critically-sized bone defects, including non-unions, cavities and segmental defects. Within the spectrum of bone grafting alternatives, autogenous cancellous bone graft is the most common treatment of non-unions (40–50%) [1–3]. Autologous bone is the gold standard in treatment of non-mineralized matrix as it is a vascularized graft that provides osteogenic cells with proper osteoinductive stimulus that enhances cell-mediated repair. However, the available amount of autologous bone is often insufficient to treat large defects and the primary alternative graft approach, cadaver bone, has clinical shortcomings ranging from risk of disease transmission to relatively poor long-term function.

Synthetic scaffolds that can recapitulate the ability of autologous bone to promote bone regeneration would therefore be of great benefit in the clinic. Such scaffolds would eliminate the need to harvest bone from patients and might allow graft properties to be tailored for individual patient needs. Unfortunately, most synthetic grafts, although osteoconductive, fall far short of the performance level of autogenous bone or cancellous allografts, as they lack proper vascularization, osteoprogenitor cells, and/or osteoinductive cues. Osteoprogenitor cells differentiate into osteoblasts and produce the bone matrix (osteoid) that later mineralizes and is remodeled into lamellar bone, hence these cells are essential for bone regeneration. Osteoprogenitor cells arise from differentiation of connective tissue progenitors (CTPs) [1,4], a heterogeneous population that includes multipotent mesenchymal stem cells (MSCs) [5–7]. Osteoinductive cues are important in synthetic grafts as they can help recruit and stimulate near-by, tissue-resident stem and progenitor cells to participate in the regeneration process. However, in many defect situations, the local environment is relatively depleted of stem and progenitor cells and thus supplementation of the graft with these essential cells is likely necessary to ensure healing.

CTPs are present in bone marrow aspirates, making marrow an attractive therapeutic source of osteogenic precursors when stem and progenitor cells for graft augmentation. Optimization of CTP isolation [4] and transplantation strategies [8–10] has led to improved bone healing in animal models [9,11–13]. However, the hypoxic, nutrient-limited, and inflammatory microenvironment of the bone wound can cause death of a substantial fraction of transplanted cells within the first few days [14–17], reducing the effective number of osteoprogenitors that contribute to the proliferative and remodeling stage of wound healing [18]. We thus hypothesize that providing bioactive cues that stimulate survival and proliferation of connective tissue progenitors within grafts, without interrupting terminal osteogenic differentiation, will improve the outcome of bone healing [1,3,18,19].

Epidermal growth factor (EGF) stimulates colony formation and proliferation of CTPs and MSCs *in vitro* without interfering with subsequent osteogenic differentiation [20–24]. EGF binds to the EGF receptor (EGFR), an essential regulator of both bone development and bone tissue homeostasis [25–27]. However, EGF drives EGFR internalization, resulting in downregulation of EGFR and ligand desensitization when cells are exposed to moderately high ligand concentrations (i.e., above the K_D_ for ligand-receptor interactions, ~1nM) [28,29].

Receptor downregulation can be avoided by presenting EGF in tethered rather than soluble form, such that it binds to the EGFR but the EGF-EGFR complex is physically restrained from internalization and downregulation by the tether that links EGF to the culture substrate. Such tethered presentation restricts EGFR signaling to the cell membrane, resulting in sustained EGFR activation and different patterns of intracellular signaling and phenotypic responses compared to soluble EGF [30,31]. MSC spreading [31] and migration [32] are enhanced with tethered compared to soluble EGF. A particularly attractive MSC phenotype elicited by tethered EGF, but not soluble EGF, is protection of MSC from pro-death hypoxia and inflammatory cytokine cues [31,33] while preserving proliferative and osteogenic potential [30,31,33,34]. This collection of beneficial effects of tethered EGF on cultured MSCs and CTPs suggests that modifying scaffolds with tethered EGF may enhance bone formation by transplanted CTPs by protecting them from death and stimulating proliferation.

Beta-tri calcium phosphate (ßTCP) scaffolds and composites thereof serve as a substrate for tissue-resident or transplanted MSCs or CTPs, evidenced by their performance in numerous animal studies [35–38] and its current use in a variety of bone void fillers in the clinic [39–41]. However, ßTCP does not have any intrinsic biological stimulatory activity and is not amenable to covalent conjugation of biomolecules. Modification of ßTCP scaffolds is thus limited to adsorption/freeze drying strategies, which often result in bolus release due to the relatively low affinity of biomolecules for the ßTCP surface.

High–affinity binding peptides, derived from combinatorial screens of peptide libraries against the surface of interest, are an appealing means to tether proteins to surfaces that lack functional groups for covalent modification [42–45]. High affinity peptides identified by screening are fused to the ligand of interest by a peptide tether, thus creating a fusion protein that binds to the surface with high affinity and presents the ligand in an accessible, bioactive form.

Here, we report the results of our screen of a commercial 12-mer phage display library to identify a high affinity ßTCP binding peptide. We used protein fusion to link the binding peptide to the human EGF domain. Further, we describe an efficient scheme to concatamerize the binding peptide and show that concatamerization up to a 10-mer increases the affinity of the EGF fusion protein for ßTCP. The bioactivity of EGF tethered to clinically-approved ßTCP scaffolds was demonstrated by assessing the plating efficiency and *in vitro* proliferative response of human MSC over 7 days in culture.

## Materials and Methods

### Fabrication of ßTCP and ßTCP-polymer composite scaffolds

**Fig 1:**
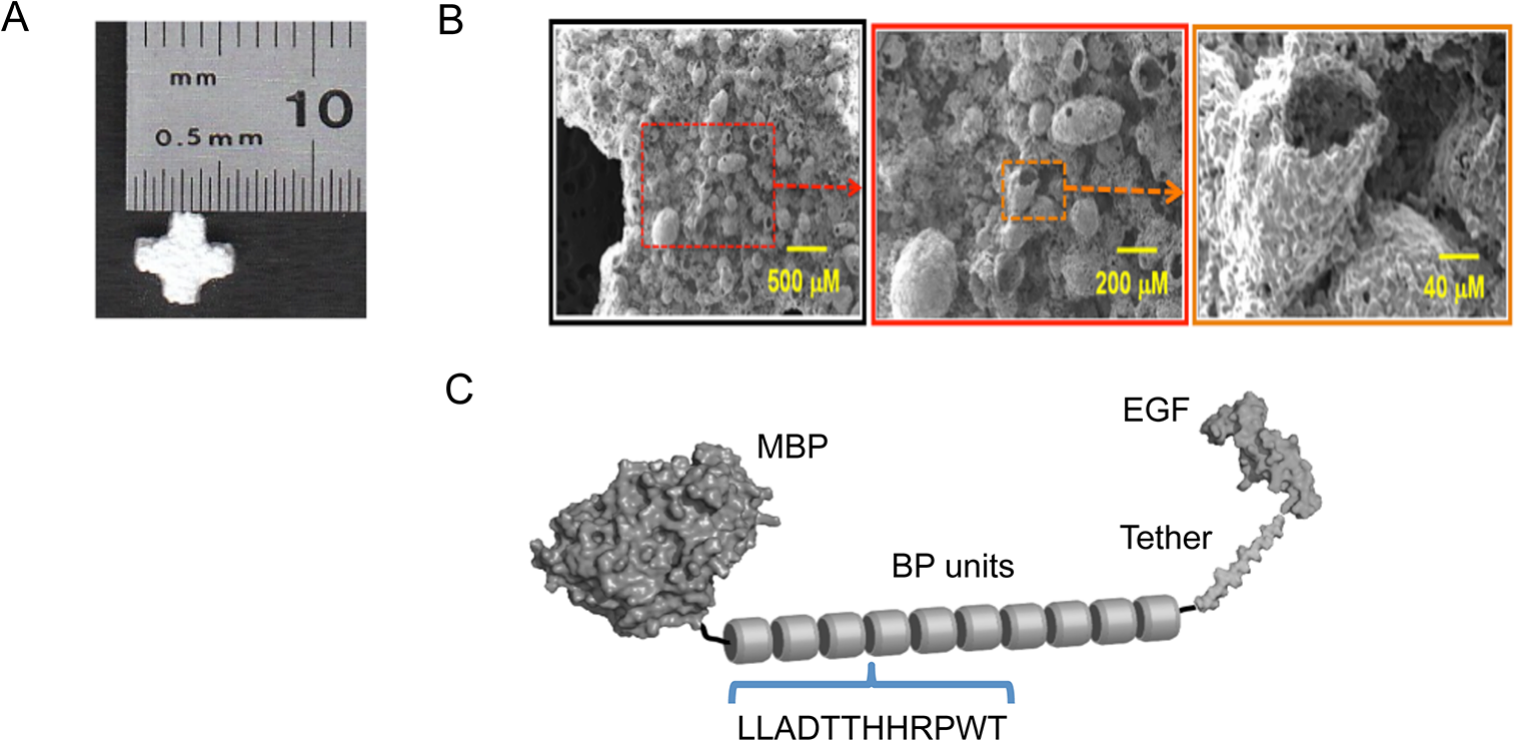
Structures of ßTCP scaffolds and EGF-ßTCP binding peptide (BP-T-EGF) fusion protein. Macroscopic appearance of a 5mm ßTCP Therilok cross-shaped scaffold used for cell culture experiments (A). These scaffolds were crushed to a coarse powder for phage panning to select binding peptides. SEM micrographs of the surface of ßTCP scaffolds at low, intermediate and high resolution as indicated by scale bars (B), Structure of the fusion protein “BP_10_-T-EGF” comprising the 12-amino acid ßTCP-binding peptide fused to EGF by flexible protease-resistant tethers flanking a coil domain (C; see S1 Table for specific sequence).

Scaffolds were fabricated at Integra Life Sciences (Plainsboro, NJ, USA) from either ßTCP or a composite of ßTCP and polylactide-co-glycolide (PLGA) using the TheriForm 3D rapid prototyping platform [46]. Briefly, to create ßTCP scaffolds, granulated ßTCP powder was sintered and sieved to <106 μm. Scaffolds were fabricated in the shape of a cross by depositing binder in a programmed sequence onto a powder bed containing a mixture of sintered ßTCP powder and porogen (spray-dried lactose powder). The scaffolds were then sintered for 20 h, dried for 1 day, leached for 2 days to remove porogen, and dried one day to yield crosses measuring 5.4x5.4x2.7mm (Fig 1A).

Scaffolds have an open porous architecture (Fig 1B) with a pore volume of about 60% porous and a mean pore diameter of 60μm (range of 5–900μm). Chemically, the scaffolds are approximately >95% ßTCP with the remaining portion being other resorbable forms of calcium phosphate. Composite ßTCP-PLGA scaffolds were fabricated in a similar fashion by mixing powders with a porogen (spray-dried lactose powder). Composite scaffolds were fused by exposure to chloroform vapor, leached, dried, and sterilized by ethylene oxide as described elsewhere [47]. The selection of size of scaffold was based on compatibility with existing tissue culture systems such as multi-well plates. The selection of geometry was made in an attempt to reduce the ratio of scaffold internal surface area to culture medium volume. A space filling shape such as a cube or cylinder would offer a higher internal surface area relative to the total culture volume in a multi-well plate. This would have led to more rapid medium depletion. A cross shape such as the one used in this study presents a lower total internal surface area and thus a lower nutritional burden per unit volume of medium. This allowed for longer culture times between medium changes (ranging between 1–3 days). A cube or cylinder geometry would have required media passages every 12–18 hours at the higher cell densities).

Scanning electron micrographs of the ßTCP scaffolds were obtained using a Jeol 5600LV Scanning Electron Microscope. Samples were mounted on stubs, sputter coated with Au/Pd (~5nm layer) and imaged at the desired magnifications using an accelerating voltage of 5 kV.

### Phage display against ßTCP scaffolds

Pure ßTCP cross-shaped scaffolds (5.4x5.4x2.7mm) from Integra Life Sciences (Plainsboro, NJ, USA) were crushed into powder, autoclaved for 35 minutes at 121^°^C and stored under dry sterile conditions prior to all experiments. The resulting sterile ßTCP powder was blocked for 24 hours at 4^°^C under moderate agitation with either sterile filtered Odyssey Blocking Buffer (OBB; non-mammalian blocking buffer; LI-COR; Lincoln, NE, USA) or 5% bovine serum albumin in phosphate buffered saline (BSA; Sigma-Aldrich, St. Louis, MO, USA). Blocked ßTCP was pelleted at 2000 RPM for 2 minutes, washed 3X with PBS then subjected to three rounds of phage display using the New England Biolabs linear 12-mer Ph.D. kit (Ipswich, MA, USA). Orthogonally blocked ßTCP (i.e. blocked with BSA vs. OBB) provided a control against panning against components of the blocking buffers. Additional controls included a ßTCP-PLGA composite cross-shaped scaffold (5.4x5.4x2.7mm), crushed into powder, similarly blocked with BSA as well as a mock tube to control against panning against tube components. After three rounds of panning, ten plaques from each of the ßTCP/BSA and ßTCP-PLGA/OBB and nine from the ßTCP/OBB block condition were picked, amplified, then sequenced (the mock condition and the ßTCP-PLGA blocked with BSA did not produce plaques after the second and third round, respectively). Sequences were analyzed for consensus using JalView Multiple Sequence Alignment Editor.

### Mutagenesis to create multimeric ßTCP-binding peptide domains in EGF fusion proteins

We had previously created a pMAL-c5X vector (New England Biolabs, Ipswich, MA, USA) expressing human EGF in fusion with various epitopes [48]. Building on this, the highest-ranked sequence from all the sequenced third round phage display panning (LLADTTHHRPWT) against ßTCP was serially cloned into a pMAL expression cassette using PCR mutagenesis and a short primer to generate a library of multimer insertions fused to EGF via protease-resistant tethers flanking a coil domain (Fig 1C). PCR mutagenesis was performed with a Quickchange Lightning II kit (Agilent, Santa Clara, CA, USA). PCR primers were designed to prime wholly within the ßTCP-binding peptide coding region thus allowing multiple insertions during a single PCR mutagenesis round. Multimer clones were sequenced to confirm DNA identity with target sequence, transformed into BL21(DE3)pLysS *E*. *coli* (Cat# 200132; Agilent, Santa Clara, CA, USA) and plated on ampicillin LB agar. We designate the final gene product produced recombinantly as BP_*n*_-T-EGF where *n* denotes the number of ßTCP-binding peptide repeats in the binding domain and *n* ranged from 1–10.

### Protein expression

Protein was expressed in 1L BL21(DE3)pLysS *E*. *coli* (Cat# 200132; Agilent, Santa Clara, CA, USA) cultures grown in ampicillin-containing (50μg/mL) luria broth (LB) at 250 rpm, 37^°^C until OD (600nm) = 0.6, then induced with IPTG and incubated at 22^°^C for 4 hours. Proteins were harvested by pelleting cultures at 3700 RPM on an Allegra G3.8 rotor at 4^°^C for 30 minutes then freezing the pellet at -80^°^C overnight followed by cell lysis using Bugbuster Reagent (EMD Millipore, Billerica, MA, USA) supplemented with PMSF and protease inhibitor cocktail (Complete Protease Mini; Roche Diagnostics Corp, Indianapolis, IN, USA). Lysed cells were centrifuged at 3700 RPM on an Allegra G3.8 rotor at 4^°^C for 1 hour. The supernatant was then diluted 1:4 in tris-buffered saline and subjected to maltose binding protein affinity chromatography in accordance with the manufacturer’s instructions (New England Biolabs, Ipswich, MA, USA). Eluted fractions were pooled and subjected to ultrafiltration through a 50,000 MWCO membrane U-tube concentrator (Novagen). The protein solution was then sterile filtered through a 0.2 μm syringe filter. Purity was confirmed by SDS-PAGE using coomassie staining. Typical purity of the full length fusion protein (73 kDa) comprising MBP, ten concatamerized ßTCP-binding peptide motifs, the tether-coil domain, and EGF, i.e., BP_10_-T-EGF, ranged from 75% to 90%, with >99% of the protein being of recombinant nature. Contaminant protein was BP_10_-T-EGF lacking the EGF motif (BP_10_-T). Similar results were observed for all concatamer lengths of binding peptide. Stock protein quantification was performed using a Nanodrop ND-2000 spectrophotometer at 280nm absorbance. The absorbance values along with the predicted extinction coefficient obtained through the ExPASy protparam tool (*http*:*//web*.*expasy*.*org/protparam*; the full protein sequence as input) were used to calculate the protein concentration. Concentrated protein stocks were used immediately or stored for a week at 4^°^C.

### Characterization of BP_n_-T-EGF binding to and elution from ßTCP scaffolds

ßTCP scaffolds or sieved pure ßTCP powder were incubated at room temperature for two hours in purified BP_*n*_-T-EGF diluted in OBB buffer. After incubation, scaffolds or powder were washed three times in three volumes of 20 mM Tris-buffered saline at pH 7.4 followed by a final wash and storage in PBS. Relative binding as a function of concatamer length and peptide concentration was determined by eluting ßTCP-bound BP_*n*_-T-EGF in 200 μL of pH 2.2 0.2M glycine buffer + 1mg/mL BSA for two hours, then analyzing the eluate by Western blot coupled with IR-dye immunofluorescence (1° Ab—Rabbit Anti-hEGF {Abcam, Cambridge, MA, USA; cat#ab9695}; 2° Ab Goat Anti-rabbit-AlexaFluor680 {Invitrogen–Life Technologies; Grand Island, NY, USA; cat# A-21109}). Control “–T-EGF” protein incorporating all elements of the BP_*n*_-T-EGF except the ßTCP-binding peptide region was used as a negative control for non-specific binding in all experiments. This negative “–T-EGF” control did not show detectable binding to ßTCP.

For the binding isotherms, 3mm and 5mm crosses were incubated with BP_10_-T-EGF protein solutions ranging from 0.2–9μM for ~36 hours at 4°C. After tethering, the crosses were washed 2x with PBS then assayed for total protein by the BCA assay. To ensure that samples were within the linear range of the BCA assay, crosses tethered with BP_10_-T-EGF at the higher end of the concentration range were divided into multiple segments and placed in individual wells of either 96-well plates or 48-well plates as follows: 0.2μM (1:1), 1μM (1:2), and 2μM (1:3) conditions for the 3mm scaffolds and the 0.4 μM (1: 2) condition for the 5mm cross were performed in 96 well plates; the 5μM condition for the 3mm cross (1:1) and the 2μM (1:1) and 9μM (1:2) conditions for the 5mm crosses were performed in 48 well plates. Water (100μL, 96 well; 400μL, 48 well) water was added to the wells with scaffolds/fragments, which were then crushed into semi-powder form (no large granules or scaffold blocks visible). The BCA reagent was then added to all wells (100μL for 96 well and 400μL for 48 well), the plates were sealed with plastic adhesive and incubated at 37°C for 50 minutes. After incubation, the plates were centrifuged for 10 minutes at 2,000 RPM at room temperature. The well plates were then gently tilted and 100μL out of each well was moved to a new 96 well plate, being careful not to take any ßTCP powder in the process. The final plate was checked to ensure that all wells were free from bubbles and the plate was read at 562nm. Background from wells without crosses or protein was used to correct for background absorbance. We confirmed that ßTCP (in scaffold or powder form) did not change the background absorbance of the BCA reagent solution. Absolute protein amounts in test wells were calculated based standard curves using BP_10_-T-EGF, where the ratio of the slope of the BP_10_-T-EGF over BSA standard curve was 1.5–1.6.

To close the mass balance, BP_10_-T-EGF protein concentrations remaining in the supernatant after the tethering process were measured via both Nanodrop (absorption at 280 nm) for relatively concentrated samples (OD > 0.3) and the BCA assay across a range of concentrations. Methods showed a strong correlation (ANOVA p-value>0.05) with no statistical differences and coefficients of variation of ~15%.

### Cell culture

Human bone marrow mesenchymal stromal cells (hBMSCs) obtained at Passage 1 (Texas A&M Health Science Center College of Medicine's Institute for Regenerative Medicine) were culture-expanded using expansion medium (EX) (αMEM with 2mM L-glutamine, 16.5% fetal bovine serum (FBS, Atlanta Biologics; Flowery Branch, GA, USA), 100 units/ml of penicillin and 100 μg/ml streptomycin) at low seeding density (50 cells/cm^2^) and frozen stocks were made when cells reached 70–80% confluency to Passage 2 (“P2”; freezing media: αMEM with 2mM L-glutamine, 16.5% FBS and DMSO mixed at a 65:30:5 v/v ratio). For the experiments, P2 stocks were expanded at low density (50cells/cm^2^) and used for experiments when cells reached 70–80% confluency.

### Validation of bioactivity of the EGF domain in fusion proteins

The intrinsic activity of the EGF domain in fusion proteins was assessed by comparing the ability of soluble BP_*10*_-T-EGF and wild-type EGF to stimulate signaling activity downstream of EGFR in hBMSC. Passage 3 hBMSCs were seeded onto Falcon 12-well tissue culture-treated polystyrene plates (Becton Dickinson & Co.; Franklin Lakes, NJ, USA) at a density of 18,000 per well and grown to a monolayer by culturing in EM. The cells were then serum starved by incubation overnight in 2% dialyzed FBS (Gibco–Life Technologies; Grand Island, NY, USA) and after overnight incubation the cells were exposed to serum-free medium for 2hr prior to experiment. The medium was aspirated and cells were exposed to serum-free media containing the corresponding ligand or negative control (no ligand) for a period of 10 minutes. The media were then aspirated, the plates were placed on ice and the wells were rinsed with ice cold 1xPBS and then incubated with ice-cold lysis buffer (Bio-Rad, Philadelphia, PA, USA) that included both protease inhibitors and phosphatase inhibitors used at 1x concentration (Complete Protease Mini and PhosSTOP phosphatase inhibitor tablets; Roche Diagnostics Corp, Indianapolis, IN, USA). While on ice, wells were scraped with rubber policemen and lysates collected and placed in the wells of a sterile, low protein binding 1.2μm filter 96-well plates (Multiscreen HTS, low protein binding, EMD Millipore, Billerica, MA, USA) and placed on top of a regular 96-well plate and centrifuged at 2,000 rpm for 5 minutes. The plate was then sealed with a microplate sealing film (Thermo Fisher Scientific; Pittsburgh, PA, USA) and stored at -80C or used immediately. The BCA assay using BSA as a standard was used to measure lysate concentrations.

Western blots for phosphor-ERK1/2 were performed by loading 10 μg of lysate into each well of an SDS-PAGE gel (Nu-PAGE Novex 4–12% Tris-bis gel, 10-well; Invitrogen–Life Technologies, Grand Island, NY, USA). Proteins in the gel were then transferred to a Nitrocellulose membrane (Bio-Rad, Philadelphia, PA, USA) using the X Cell II blot unit (Invitrogen–Life Technologies; Grand Island, NY, USA). The membranes were blocked using OBB for 1hr at room temperature (RT). After the blocking step, the blots were incubated overnight with the primary antibodies (1° Ab) targeting phospho-ERK1/2 (Rabbit anti-phospho-ERK1/2 (Thr202/Tyr204) Ab cat #4370; from Cell Signaling in Danvers, MA, USA) and the loading control GAPDH Ab (Mouse anti-GAPDH Ab, cat#AB9484; from Abcam in Cambridge, MA, USA) diluted in 1xPBS containing 0.1% Tween-20 (T-20). The p-ERK1/2 antibody was diluted 1:1000 and the GAPDH antibody was diluted 1:2000 (stock is 1mg/mL; use at 0.5–1 μg/mL). After 1° Ab incubation, the blots were washed 4x with 1xPBS containing 0.1% T-20 and incubated with the secondary antibody (2° Ab) (1:10,000 dilution) tagged with IR-range dyes (700-800nm; Goat anti-mouse-IR800 dye from LI-COR; Lincoln, NE, USA, and Goat anti-rabbit-AlexaFluoro680 from Invitrogen–Life Technologies; Grand Island, NY, USA) in PBS with 0.1% T-20 and 0.01% SDS. The blots were then incubated in the Ab solutions for 45 minutes at RT with gentle shaking. Blots were washed 4X, placed in neat 1xPBS and scanned at in a LI-COR machine (700nm and 800nm channels). The intensities from each band (p-ERK1/2 at 700nm or GAPDH at 800nm) were measured using the Odyssey 3.0 software (LI-COR; Lincoln, NE, USA). All the intensity values for p-ERK1/2 were normalized to the GAPDH loading control and then normalized to the no EGF controls (negative control). Results from all blots (N = 3) were then averaged and plotted. For the westerns against the EGF domain of BP_n_-T-EGF, a similar procedure was performed but using a Goat Anti-hEGF antibody (R&D systems, Minneapolis, MN, USA; cat# AB-236-NA) as the 1° Ab and a Donkey Anti-Goat IRdye680RD (LI-COR; Lincoln, NE, USA; cat# 926–68074) as the 2° Ab.

### hBMSC proliferation assays on ßTCP scaffolds

Cell proliferation was determined using the Alamar Blue (AB) assay (Invitrogen–Life Technologies; Grand Island, NY, USA) at 7 days post-seeding. Passage 3 hBMCSs were seeded at 2,000 cells per well onto 5mm ßTCP cross-shaped scaffolds (5.4x5.4x2.7mm; Therilok from Integra Life Sciences, Plainsboro, NJ, USA). Cells in media were seeded into the center of the well directly on top of the crosses (200uL total). This resulted in a seeding fraction of ~16–20% of total cells seeded for 3mm crosses and ~31–42% for 5mm crosses, approximately comparable to the cross sectional area of the crosses in the well. This agrees well with previous observations that static seeding generally leads to low seeding densities unless the scaffold occupies most of the well surface [49].

Following seeding, cells were cultured overnight in EX media. Crosses were then moved to new wells to eliminate contributions from cells that had attached to the bottom of the well. The crosses were then cultured in new plates for a cumulative time of 7 days in EX media. Each condition was performed in triplicate. After the 7-day culture the cell-containing ßTCP scaffolds (EGF-tethered and controls) were moved into a sterile, low-protein binding 1.2μm filter 96-well plate (Multiscreen HTS, low protein binding; EMD Millipore, Billerica, MA, USA) and the Alamar Blue reagent (Invitrogen–Life Technologies; Grand Island, NY, USA) was then added to each well (mixed in a 1 part of the 10x AB reagent to 1 part EX media according to the manufacturer’s instructions). The plate was then incubated at 37°C, 5% CO_2_ for 4 hours with gentle mixing. After incubation, the filter plate unit was attached on the bottom to a flat-bottom 96-well plate and centrifuged at 1000 rpm for 3 minutes. This method allowed for complete recovery of AB dye-media mixture. One-hundred microliters of the resulting AB dye-media mixture collected was transferred to a new flat-bottom 96-well plate to be read by a SpectraMax M2e multi-well fluorescent plate reader (Molecular Devices Corp.; Sunnyvale, CA, USA) at a 570 nm excitation wavelength and 585 nm emission wavelength as recommended by manufacturer’s instructions. The background fluorescence reading (AB solution in media with a cell-free ßTCP scaffold) was subtracted from the AB fluorescence reading for each well to obtain the net fluorescence. The average net AB fluorescence units from each condition (N = 3 per condition) were normalized to no EGF ligand condition (control).

### Analysis of plating efficiency on ßTCP scaffolds

The Alamar Blue assay is relatively insensitive at low cell numbers. Hence to determine the plating efficiency of cells on control and tethered EGF crosses at early time points (12 and 24 hr), we developed a protocol to directly count cells that were fixed and DAPI-stained by embedding crosses in agarose followed by a demineralization step to dissolve the cross, allowing direct microscopic observation of the cells in situ. In these experiments, we used smaller ßTCP cross-shaped scaffolds (dimensions: 3.6x3.6x1.8mm, Therilok II, Integra Life Sciences, Plainsboro, NJ, USA) in order to have enough working distance to directly count all cells adhered onto scaffolds (with 10x objective) using a demineralization protocol. Crosses were fabricated by a process identical to that used for larger crosses, sterilized by autoclaving, and then were treated according to the EGF tethering protocol described above, using PBS as a control.

Crosses were placed in individual wells of a 96-well plated and seeded with 11,000 cells in 200uL of EX media leading to ~1,700–2,200 cells landing on the 3mm scaffold (based on predicted ~16–20% seeding efficiency for 3mm crosses placed in 96-well plates). Following culture for 12 or 24 hours in EX media, cells were rinsed with 1x PBS, fixed for 2 hours with 2% glutaraldehyde/2% formaldehyde in 1x PBS, then washed with multiple rinses in 1xPBS (3x; 30 seconds each). Crosses were then embedded in agarose, demineralized in a mild HCl solution and left at 4C until embedded scaffolds appeared translucent. The HCl solution was then neutralized by multiple rinses with 1x PBS. The cells within the embedded scaffolds were stained with DAPI and then imaged under a confocal microscope. Stacks were taken using a 10x objective using the DAPI filter. Z-stacks covered a z-range of 1.8mm (ßTCP scaffold height = 1.6mm) and an XY Z-stack sweep covered a total area of ~4x4mm range on the XY axis (total area scanned 4.4x4.4x1.8mm). Stacks along the XY axes covering the complete cross were acquired and cells in each stack were counted using the DAPI signal and the spots function in the IMARIS imaging software (Bitplane; South Windsor, CT, USA).

## Results

### ßTCP binding peptides identified by phage display

**Fig 2:**
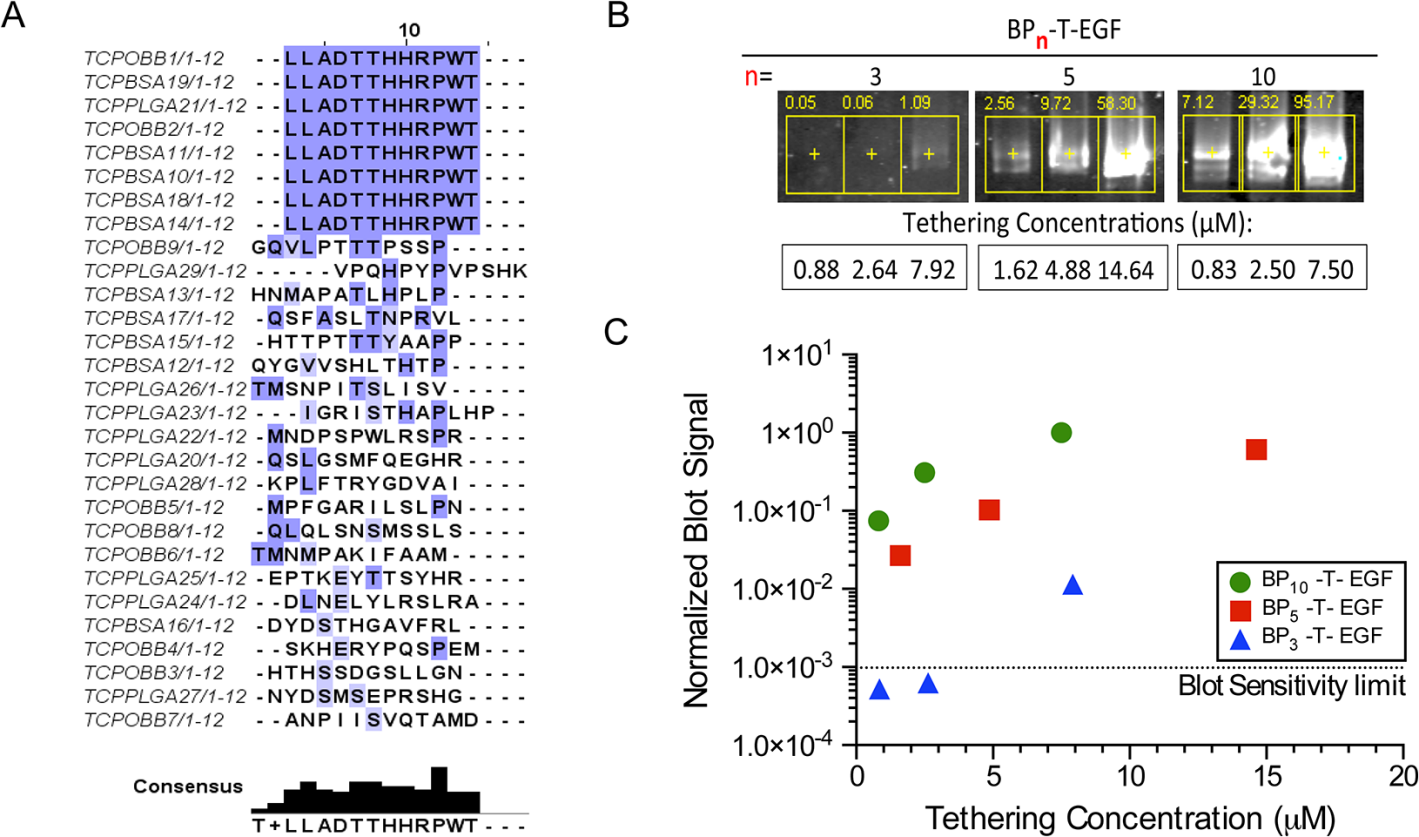
Identification of ßTCP-binding peptide and concatamerization of the sequence in EGF fusion proteins. Sequence alignment of multiple panning experiments against ßTCP material using orthogonal blocking and panning against composite ßTCP-PLGA substrates showing that the consensus sequence in 8 of 29 clones is LLADTTHHRPWT (A). Anti-EGF immunoblot performed against scaffold-eluted BP_*n*_-T-EGF fusion protein with different binding peptide repeats (*n* = 3, 5 and 10) tethered at dilutions as indicated, illustrating greater tethering with increase *n* repeats of the binding peptide in the concatamer (B). Quantification of anti-EGF immunoblot signals depicted in 2B shows that the 10-mer repeat of the 12-amino acid ßTCP-binding peptide imparts the highest affinity binding to the BP_*n*_-T-EGF fusion protein (C).

Three rounds of panning yielded plaques for three of the six conditions: ßTCP blocked with BSA; ßTCP blocked with OBB buffer (non-mammalian blocking buffer); and ßTCP-PLGA composite blocked with OBB buffer. Mock conditions (tubes only) and ßTCP-PLGA blocked with albumin (BSA) did not yield plaques at the 2^nd^ and 3^rd^ round respectively. The sequence Leu-Leu-Ala-Asp-Thr-Thr-His-His-Arg-Pro-Trp-Thr was identified in a total of 28% (8/29) of the clones: 5 from ßTCP blocked with BSA; 2 from ßTCP blocked with OBB protein buffer and 1 from composite ßTCP-PLGA blocked with OBB buffer, (Fig 2A). The remaining 21 clones showed only modest sequence similarity based on the BLOSUM62 scores (Fig 2A).

The 12-amino acid consensus sequence (M_w_ = 1448 Da) includes one negatively charged residue (Asp), one positively-charged residue (Arg), and has a predicted pI of 6.92. Interestingly, the sequence includes two histidines (nominal pK of 6.1), which may become protonated in the low-pH environment of post-surgical inflammation or abstract protons from the calcium phosphate surface. The peptide is predicted to be relatively soluble based on a grand average of hydropathicity (GRAVY) score in the moderately negative range (-0.800). The extinction coefficient (water) at 280 nm was determined to be 5500 M^-1^ cm^-1^.

### Binding affinities of EGF fusion proteins with single and concatameric ßTCP binding peptides

Based on the biophysics of interactions between the EGFR and tethered EGF, it is desirable to present the EGF moiety using a spacer to enhance accessibility of the ligand [31,50,51]. We therefore fused the binding peptide sequences to the N-terminus of human EGF (53 amino acids; MW = 6.2KDa) with an intervening 106 amino acid sequence comprising a coiled-coil sequence (46 amino acids, MW = 5.4 KDa) flanked on both ends by a flexible, protease-resistant spacer (25 amino acids; MW = 1.9KDa) along with several restriction enzyme sites for cloning (See S1 Table for protein sequence). In previous work, we used paired high-affinity heterospecific coiled-coil sequences with the same protease-resistant spacer in order to dimerize EGF and other EGFR family ligands, and had determined that the fusion proteins and their dimers were active when constructed as either N-terminal or C-terminal fusions [48].

Further, we reasoned that the binding affinity of the peptide to ßTCP might be further enhanced by concatamerization of the binding sequence. We used mutagenesis (see Methods) to concatamerize the 12-mer ßTCP binding peptide, yielding protein fusions with 3, 5, and 10 repeats of the 12 amino acid ßTCP binding unit (LLADTTHHRPWT) flanked by other relevant protein domains as depicted in Fig 1 (see Methods). We first examined the relative binding affinities of the fusion proteins as a function of the number of repeats of the binding domain in the fusion protein using a semi-quantitative approach based on eluting proteins followed by western blot analysis (Fig 2B and 2C). We titrated the adsorption concentrations across a range of 0–15 μM and found that binding exhibited a profound dependence on the number of 12-mer repeats (3, 5 or 10) in the binding domain (Fig 2B and 2C). Based on these results, we selected the fusion protein with the 10x linear concatamer, referred to as BP_10_-T-EGF (See Fig 1), to perform all subsequent cell interaction experiments.

### BP_10_-T-EGF in solution exhibits wild-type soluble EGF activity

**Fig 3:**
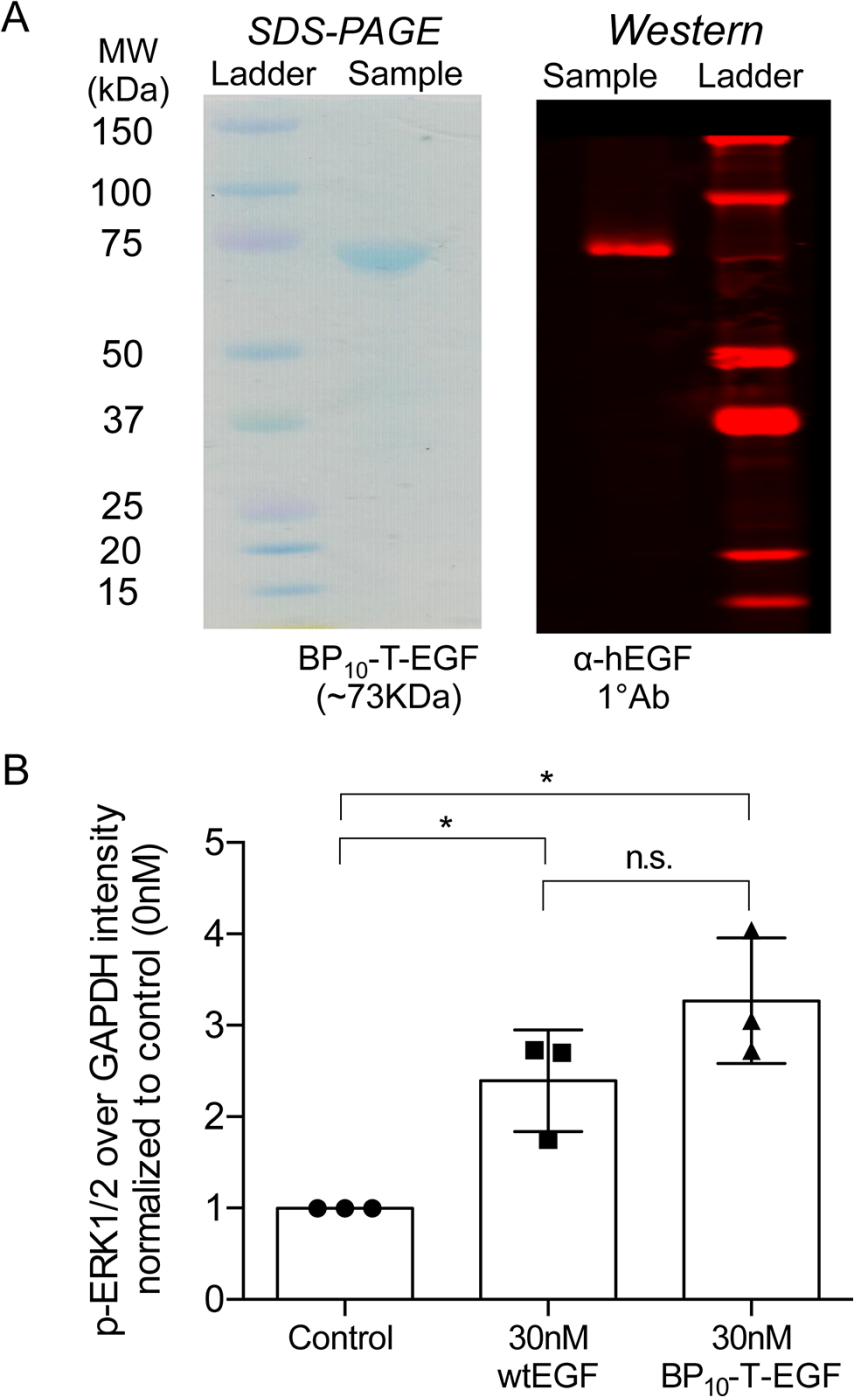
BP_10_-T-EGF fusion proteins activate EGFR. Coomasie blue staining of purified BP_10_-T-EGF by SDS-PAGE shows a single predominant band at the expected molecular weight (73 KDa), and this band contains the EGF immunoreactive activity (A) Soluble BP_10_-T-EGF elicits phosphorylation of ERK 1 and 2 in a comparable fashion to wild type EGF (B).

After selecting BP_10_-T-EGF as the best binder, we confirmed the purity and activity of each recombinantly-produced 10-mer protein batch prior to use in cell phenotypic assays. Western blots of samples subjected to SDS-PAGE showed that the EGF is co-localized with the 73 kDa band (Fig 3A), as expected for intact BP_10_-T-EGF. Biological activity of BP_10_-T-EGF compared to control wild type EGF was assessed by analyzing activation of Erk-1 and Erk-2 (Erk1/2), a signaling pathway that shows maximal phosphorylation 7–15 min after stimulation of EGFR in MSC [20,30,31,50–53]. Compared to unstimulated controls, a 2.5 to 3-fold increase in ERK1/2 phosphorylation was observed 10 min after stimulation of MSC by either wild type EGF or BP_10_-T-EGF (Fig 3B). Results were normalized to the loading control GAPDH (N = 3 per condition; one-way ANOVA p<0.0001; *pairwise comparisons with control were p≤0.001 using Tukey’s multiple comparisons test; all data was log-transformed before analysis). There was no statistical difference between the pairwise comparisons of wild type EGF and soluble BP_10_-T-EGF (p>0.05; Tukey’s test). Thus, the EGF domain in BP_10_-T-EGF appears to be fully competent to activate the EGFR.

### Binding and release of BP_10_-T-EGF tethered to ßTCP scaffolds

**Fig 4:**
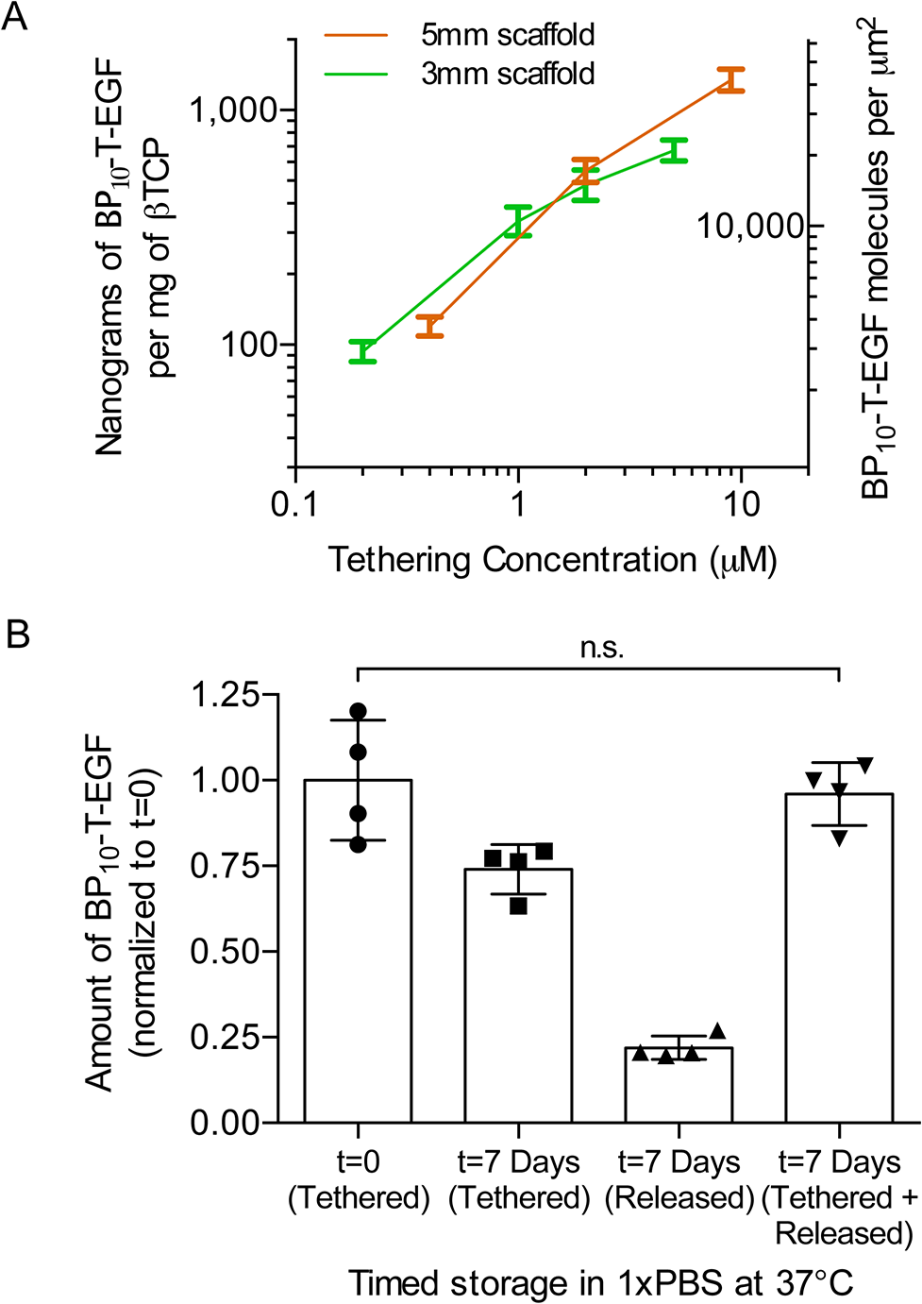
Binding of BP_10_-T-EGF to ßTCP scaffolds and elution over 7 days. Binding isotherms of *BP*_*10*_ *-T-EGF* for 5mm and 3mm crosses (A). Analysis of released and bound BP_10_-T-EGF protein for the 2μM tethering condition shows that assays are robust (i.e., the combined bound and released at 7 days is statistically indistinguishable from the initial amount) and that 75% of the BP_10_-T-EGF fusion protein remains bound to the scaffold after a 7-day incubation in 1xPBS at 37^°^C (B).

Comparable binding isotherms for BP_10_-T-EGF were observed for crosses of 3 mm and 5 mm using concentrations of 0.2–9 μM soluble protein (Fig 4A). The resulting range of tEGF surface densities was estimated as 4,000–45,000 EGF/μm^2^ (see Methods), well within and above the value of 500–5,000 EGF/μm^2^ found to provide maximal stimulation to epithelial and mesenchymal cells in previous studies employing EGF tethered to polymer substrates [31,50,51]. However, because these previous studies employed tethering schemes that fostered local clustering of tethered EGF, and the binding peptide approach would not necessarily lead to such localized clustering, a tethering concentration of 2 μM (~ 10,000 EGF/μm^2^) was chosen for further studies. After a 7-day long incubation of treated (2 μM) scaffolds in 1xPBS at 37C, a ~25% release of tethered BP_10_-T-EGF protein was observed (N = 4 per condition, Fig 4B). Another stability experiment performed at lower temperatures (4C) using the same buffer revealed there was no statistically significant release of BP_10_-T-EGF protein from 3mm ßTCP scaffolds (Normalized protein amounts were 1.02 +- 0.09 (day 0) and 1.02 +- 0.11 (day 5); both were normalized to t = 0; N = 3 per condition).

### Tethered EGF stimulates an increase in hBMSC number on scaffolds following 7-day culture

After establishing that the EGF domain of the BP_10_-T-EGF fusion protein induced bioactivity when it was used in soluble form for MSC stimulation (activation of signaling pathways downstream of activated EGFR), we investigated phenotypic responses of low passage primary hBMSC cultured on ßTCP scaffolds modified with BP_10_-T-EGF fusion protein for three different densities of adsorbed BP_10_-T-EGF fusion protein. We have previously shown that EGF tethered to polymer substrates via polyethylene oxide (PEO) tethers can enhance proliferation of hBMSC maintained in both expansion and osteogenic media [34] We used day 7 as a metric for comparison in order to allow for several MSC population doublings [54].

**Fig 5:**
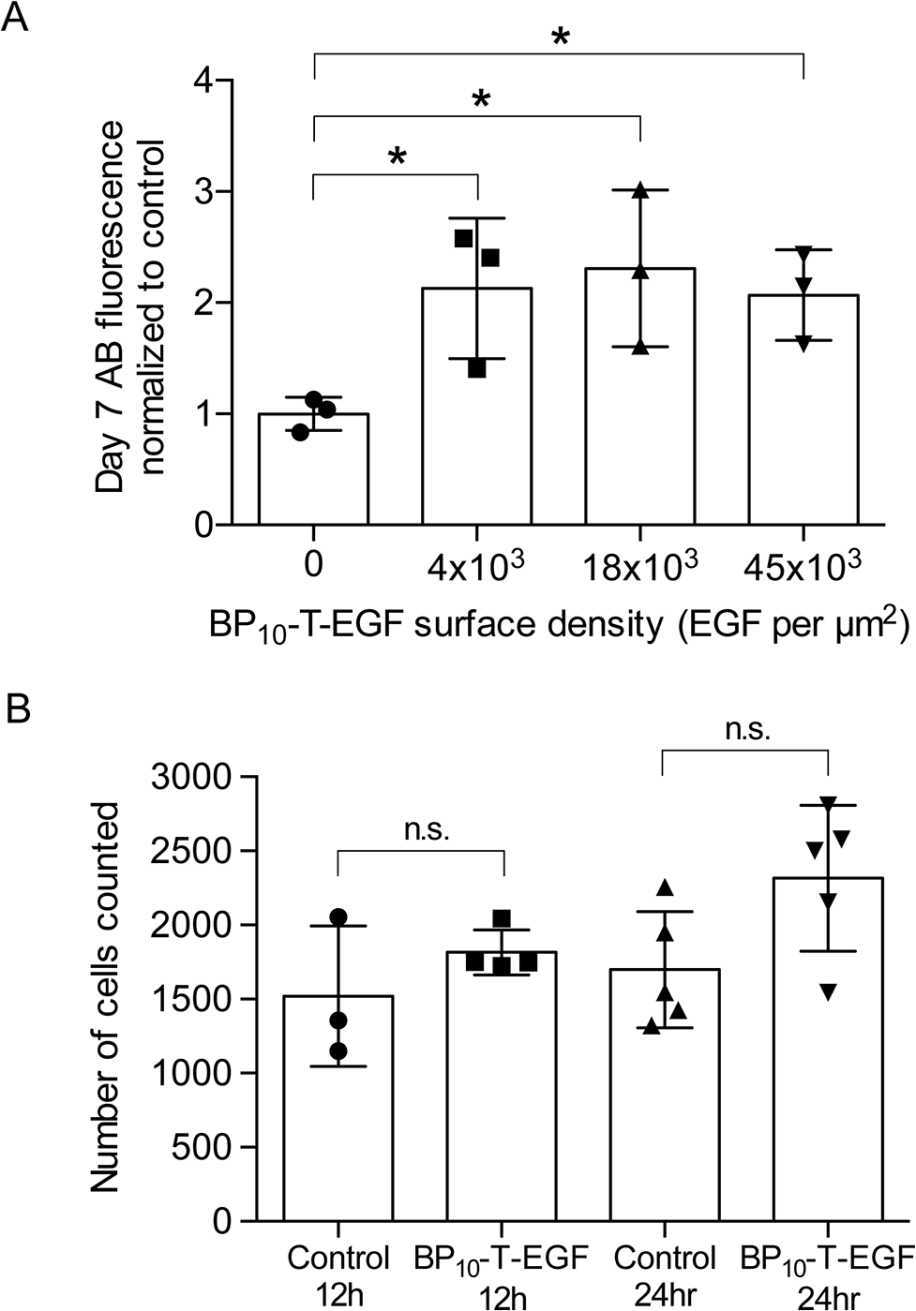
Tethered BP_10_-T-EGF induces in vitro expansion of hBMSCs cultured on ßTCP scaffolds. Cells were seeded on control and BP_10_-T-EGF- treated scaffolds, cultured in expansion medium for 7 days and then subjected to Alamar blue assay to assess cell numbers relative to controls. A 2–2.3 fold increase in total hBMSC number was observed across three different tethered BP_10_-T-EGF surface densities (N = 3 per condition; one-way ANOVA p-value<0.05 (p-value = 0.02); all pairwise p-values of tethered EGF vs control were <0.05 using Tukey’s multiple comparison test) (A). To parse effects that tethered EGF might have on plating efficiency, MSCs were cultured on ßTCP scaffolds for 12–24 hr and the number of attached cells was then determined using a visual count of nuclei following acid demineralization of agarose-embedded scaffolds. Results from 12-hr and 24-hr cell culture revealed no statistical difference (n.s.; N≥3 per condition; One-way ANOVA p-value>0.05) in the number of hBMSCs as a function of condition, indicating that tethered EGF is not altering the plating efficiency (B).

Scaffolds (ßTCP crosses, see Methods) were pre-incubated with BP_10_-T-EGF solutions at concentrations of 0.4 μM, 2 μM, and 9 μM in order to achieve a range of surface densities (estimated as 4,000–45,000 BP_10_-T-EGF per μm^2^) and to determine dose response. Human BMSCs were seeded onto the treated and control scaffolds and cultured for 7 days in expansion medium. After 7 days, the relative cell numbers were quantified using the Alamar Blue reagent, using cells seeded on standard plates at different densities as a calibration to ensure the assay was in the linear range. All scaffolds treated with BP_10_-T-EGF had a 2–2.3 fold greater number of hBMSC number compared to surfaces without BP_10_-T-EGF (Fig 5A; N = 3 per condition; one-way ANOVA p-value<0.05 (p-value = 0.02); all pairwise p-values of BP_10_-T-EGF vs control were <0.05 using Tukey’s multiple comparison test). No statistical differences were observed between the different BP_10_-T-EGF surface densities (All pairwise p-values>0.05 using Tukey’s test). These results indicate that EGF-tethered onto ßTCP scaffolds does not impair expansion of hBMSCs, as the final cell number was greater than the initial number (data not shown), but these data are not sufficient to conclude that tethered EGF enhances proliferation, as differences in initial plating efficiencies together with comparable expansion rates may account for the observed differences at day 7. To parse these mechanisms, we next examined plating efficiencies.

### Tethered EGF does not alter plating efficiency of hBMSCs seeded on ßTCP scaffolds

The Alamar Blue assay and other similar kinds of proliferation assays do not have sufficient sensitivity to detect the relatively small number of cells present on scaffolds immediately after seeding. Hence, we developed an approach to directly count cells seeded on scaffolds by embedding scaffolds in agarose, demineralizing to reveal an optically-clear mold, then staining cells and observing them with confocal microscopy. Using this method, we found no statistical differences between the direct count of P3 hBMSCs in the control or BP_10_-T-EGF conditions for both the 12hr and 24hr time point (Fig 5B). This suggests that the increase in hBMSC number observed after a 7-day culture under BP_10_-T-EGF conditions was most likely due to induction of proliferation and not due to differential plating efficiency.

## Discussion

Here we demonstrate the use of a ßTCP-binding peptide discovered through phage display and concatamerized to improve its affinity, to reliably tether EGF onto ßTCP scaffolds for stimulation of hBMSCs. This high-affinity, concatamerized binding peptide domain is of moderate size (120 amino acids, ~14 kDa), and does not contain unnatural amino acids or post-translational modifications like other ceramic or apatite binders [55–57], making it compatible with standard protein production and manufacturing strategies. This facile tethering approach only requires incubation of the fusion protein (see Fig 1) with the ßTCP substrate and subsequent washings to remove excess unbound protein. This approach thus allows for easy modification of ßTCP bone void filler scaffolds with bioactive ligands that can enhance the survival, proliferation or differentiation of tissue-resident or transplanted osteoprogenitors (i.e. MSCs and CTPs; [4,7,8,18,58].

We focused on EGF as the fusion partner for the ßTCP-binding peptide motif because of the multifaceted roles EGFR plays in bone development and homeostasis in vivo, together with previous studies demonstrating that EGF exerts beneficial effects on migration, colony formation, and, proliferation of bone marrow-derived CTPs when covalently tethered to model polymer surfaces [31–34,51]. Simple adsorption of EGF to the scaffold is not a reliable approach for bioactive delivery of EGF for this application. We could not detect binding of EGF fusions lacking the ßTCP-binding domain, an observation consistent with reports that wild-type EGF does not bind hydroxyapatite [57], a material with composition and surface charge similar to ßTCP [59], likely due to repulsion as both possess negative surface charges around the same order of magnitude [59,60].

We observed retention of ßTCP-bound BP_10_-T-EGF bioactivity across a range of surface densities (estimated as 4,000–45,000 EGF/μm^2^) as assessed by enhancement of MSC proliferation (Fig 5A). We investigated this range based on previous reports that tethered EGF enhances survival, proliferation, and colony formation by MSCs and CTPs when tethered to polymer surfaces at densities of ~5,000 EGF/μm^2^ [31–34,51]. We did not observe a statistically-significant effect of tethered EGF density on proliferation in the range of 4000–45,000 EGF/μm^2^, but this is not surprising as tethered EGF densities as low as ~400 tethered EGF/μm^2^ induce maximal DNA synthesis in primary hepatocytes and low as ~700 tethered EGF/μm^2^ induces maximal response in fibroblasts and keratinocytes [50,57,61]). We note that we attribute the increase in cell number to proliferation rather than enhanced initial attachment, as assessment of cell numbers present on the scaffolds by direct visual counting of nuclei at 12 and 24 hr post-seeding showed no statistical difference among conditions.

Although we did not directly measure the release and consumption of tethered EGF in the presence of cells, the finding of enhanced proliferation over 7 days together with observations that 75% of the protein was still retained after 7 days of incubation in PBS at 37ºC (Fig 4B) suggest that the tethered protein remains active for at least several days in culture. There are two primary mechanisms of BP_10_-T-EGF release. ßTCP exhibits small but non-zero solubility over a broad range of pH values, thus some small amount of BP_10_-T-EGF release is expected to occur due to surface dissolution of the ßTCP (solubility limit at ~0.0005 g/L as reported by [62]). However, by calculating the solubility limit at 200uL (volume of 96 well plates, corresponds to 0.1 micrograms of ßTCP) and using both the surface area of the scaffolds (0.244 m^2^/gram; Hg porosimetry) and the initial BP_10_-T-EGF surface density (~19 micrograms at 2uM tethering), we estimate that only about 100 picograms of protein are released due to surface dissolution. Since the amounts we tether are in the microgram range, we believe transient unbinding of the concatemer region from the surface of ßTCP coupled to extracellular factors play a bigger role in release.

The success of using a filamentous phage display library to obtain high affinity binders is not assured, as material properties, panning protocols, peptide length and library size can be limiting factors. However, reasonably high affinity binders to a variety of surfaces, including polymers, metals and, ceramics have been identified through commercial and non-commercial libraries [42–44,63–69]. Variations in display strategies include the number of peptides presented per phage, cyclized vs linear presentation, and peptide lengths [42,70,71]. Each mode of presentation has advantages and disadvantages with respect to library size, valency of display, and structural flexibility of peptides. Due to the polyvalent nature of M13 p3 and p8 phage display, avidity can play a role in binding as p3 display offers 3–5 copies of the peptide and p8 display offers ~2700 copies [72–75]. This polyvalency can lead to a higher apparent affinity of the phage clones due to avidity, which leads to reduced binding once the monovalent peptides are cloned and expressed.

Typical binder affinities of monovalent peptides from panning experiments with commercial kits tend to be in the low μM to mid nM range [44,76–78]. Typically, 3 to 5 rounds of screening are sufficient to obtain binders with desired affinities [42–44,68]. Additional rounds (>4) may introduce phage-specific factors (e.g. phage replication) that reduce the sequence diversity with minimal increase in affinity of the peptide pool [79,80]. After 3 rounds of panning against ßTCP using the 12-aa p3 M13 phage display kit (see Methods) we obtained 9–30 plaques per panning condition, except on tube controls which did not yield plaques at round 3. After sequencing 9–10 phage plaques per condition, we found a consensus ßTCP binding sequence (LLADTTHHRPWT) that was present in 8 out of 29 clones. Importantly, this consensus sequence emerged across orthogonal ßTCP blocking conditions (BSA and OBB) as well as panning against a composite of ßTCP with the degradable polyester biomaterial PLGA, suggesting it is highly specific for the target. We then created a fusion protein comprising this 12 amino acid peptide fused to human EGF via protease-resistant spacer flanking a coil-coil motif [48] and expressed the protein with a MBP motif to increase solubility of the purified final protein product. This first generation fusion protein did not exhibit the desired high affinity binding to ßTCP, presumably because the 12 amino acid peptide alone could not offset the thermodynamic driving force for the 73,000 dalton molecular weight protein (including the MBP domain) to remain soluble.

We thus exploited a polyvalency approach in which created a linear concatamerization of the binding peptide to increase the driving force for surface adsorption. Sequential increases of the number of 12-mer binding peptide sequences in the concatamer, from 3 to 5 to 10, were associated with increased affinity of the fusion protein with the ßTCP surface (Fig 2B and 2C). The repeat number is representative of the lower and higher bounds of p3 peptide copies per phage (i.e., 3- and 5- mer [72–74]), while the 10-mer is a greater valency than the p3 display could theoretically offer. Linear concatamerization has previously been used with some success to increase binding peptide affinity [68], but is not a general approach that works with all peptides tested. Intuitively, the domain spacing must align with multiple repeats of the affinity target on the surface in order to maintain multiple high affinity contacts. We did not systematically investigate the role of domain spacing by varying the linker length between concatenated peptide sequences, but speculate that further improvements in affinity could be achieved by changing this variable.

Many features of the 12 amino acid binding sequence we identified are consistent with affinity for calcium phosphate. Portions of the LLADTTHHRPWT sequence serve as common motifs found in proteins with affinity for binding to phosphate-containing compounds (i.e. ATP/ADP/AMP/UDP, Phosphoglyceric acid (PGA), DNA and tRNA, among others), with 13 out of the top 15 hits on a BLAST search illustrating such binding capabilities or ability to bind to divalent calcium. Amino acids present in the mid-portion of our peptide (DTTHHR)—including aspartic acid, threonine, histidine, and arginine—are all capable of forming hydrogen bonds and were prominently implicated in the interactions with phosphate groups within the active site of several of the enzyme families from the BLAST results (PDB ID#s: 1JJV, 4I1V, 4DEC, and 3V4R; [81,82]). The TT motif has been implicated in the interactions of dephospho-CoA kinases with the diphosphate group of adenosine di-phosphate (ADP) through hydrogen bonding mechanisms (PDB ID#s: 1JJV, 4I1V; [83]), suggesting a potential role for the TT motif in our binding peptide for interaction with calcium phosphate. Further, amino acids with basic side or acidic chains like arginine and aspartic acid can form salt bridges with the phosphate and calcium ions, respectively. Aspartic acid residues, along with glutamate and carboxy-glutamate residues in proteins are known to be able to form coordination complexes with calcium ions in solution [84]. There are several hydroxyapatite (HA) and calcium phosphate binding peptides that contain streaks of or domains rich in acidic amino acids [85–89]. Some of these are based on binding domains from proteins found in bone matrix like bone sialoproteins, osteonectin, among others [86,87,90–92]. Histidine residues can coordinate with divalent cations like Ca^+2^ [81], and both histidine and aspartic acid residues have been speculated to accelerate nucleation of calcium phosphates (CaP) by removing protons from phosphate groups during the hydrogen bonding reaction [93].

We speculate that the central 6aa polar domain in the binding peptide dominates the energetics of interactions with the surface, with the flanking hydrophobic amino acids (LLA on N-terminus; and PW on the C-terminus) providing some conformational support or shielding water molecules away from the surface to strengthen surrounding hydrogen bonds. However, mutational analysis would be required to precisely detail the role of individual or group of amino acids. Such analysis in other systems often reveals that only a few amino acids are crucial for binding, and the surrounding amino acids often provide support (structural or chemical) to optimize the interactions of those critical residues with the surface [44,45,66,69,94,95].

Taken together, these results provide compelling motivation for ongoing efforts to assess the colony-forming performance of BP_10_-T-EGF scaffolds with human bone marrow samples, and to translate the *in vitro* findings to in vivo bone healing models employing transplants of aspirated marrow, such as those in the canine femoral defect model [47]. Future studies might also address alterations in the protein design to improve expression and purification properties.

## Conclusion

We showed that a phage display-derived, concatamerized ßTCP binding peptide comprising 12 amino acids is sufficient to tether EGF onto ßTCP scaffolds in a stable manner in a fusion protein construct, and that the tethered EGF is active as assessed by MSC proliferation. The concatamerized binding peptide shows high-affinity towards ßTCP and can be used to tether other growth factors or bioactive ligands. This provides a versatile foundation for controlled, single or multi-protein delivery on ßTCP-based bone grafting materials.

## Supporting Information

S1 Table BP_10_-T-EGF full protein sequence. (DOCX)

## References

[1] Greenwald S, Boden SD, Barrack RL, Bostrom MP, Goldberg VM, Yaszemski MJ, et al. The evolving role of bone-graft substitutes. In: American Academy of Orthopedic Surgeons. 77th Anual Meeting [Internet]. 11 Aug 2010 [cited 10 Aug 2014]. Available: http://www.aatb.org/aatb/files/ccLibraryFiles/Filename/000000000322/BoneGraftSubstitutes2010.pdf.

[2] Giannoudis PV, Dinopoulos H, Tsiridis E. Bone substitutes: an update. Injury. 2005;36 Suppl 3: S20–7. 10.1016/j.injury.2005.07.029 16188545

[3] Roberts TT, Rosenbaum AJ. Bone grafts, bone substitutes and orthobiologics: The bridge between basic science and clinical advancements in fracture healing. Organogenesis. Landes Bioscience; 2012;8: 114 10.4161/org.23306 23247591 PMC3562252

[4] Muschler GF, Boehm C, Easley K. Aspiration to obtain osteoblast progenitor cells from human bone marrow: the influence of aspiration volume. J Bone Joint Surg Am. 1997;79: 1699–1709. 9384430 10.2106/00004623-199711000-00012

[5] Dominici M, Le Blanc K, Mueller I, Slaper-Cortenbach I, Marini F, Krause D, et al Minimal criteria for defining multipotent mesenchymal stromal cells. The International Society for Cellular Therapy position statement. Cytotherapy. 2006;8: 315–317. 10.1080/14653240600855905 16923606

[6] Majors AK, Boehm CA, Nitto H, Midura RJ, Muschler GF. Characterization of human bone marrow stromal cells with respect to osteoblastic differentiation. J Orthop Res. 1997;15: 546–557. 10.1002/jor.1100150410 9379264

[7] Pittenger MF. Multilineage Potential of Adult Human Mesenchymal Stem Cells. Science. 1999;284: 143–147. 10.1126/science.284.5411.143 10102814

[8] Patterson TE, Kumagai K, Griffith L, Muschler GF. Cellular strategies for enhancement of fracture repair. The Journal of Bone & Joint Surgery. 2008 ed. 2008;90 Suppl 1: 111–119. 10.2106/JBJS.G.01572 18292365

[9] Muschler GF, Matsukura Y, Nitto H, Boehm CA, Valdevit AD, Kambic HE, et al. Selective retention of bone marrow-derived cells to enhance spinal fusion. Clin Orthop Relat Res. 2005;: 242–251. 10.1097/01.blo.0000149812.32857.8b PMC1425153 15738828

[10] Bruder SP, Kraus KH, Goldberg VM, Kadiyala S. The effect of implants loaded with autologous mesenchymal stem cells on the healing of canine segmental bone defects. J Bone Joint Surg Am. 1998 ed. 1998;80: 985–996. 9698003 10.2106/00004623-199807000-00007

[11] Gupta MC, Theerajunyaporn T, Maitra S, Schmidt MB, Holy CE, Kadiyala S, et al Efficacy of mesenchymal stem cell enriched grafts in an ovine posterolateral lumbar spine model. Spine. 2007;32: 720–6– discussion 727. 10.1097/01.brs.0000258863.40984.32 17414903

[12] Brodke D, Pedrozo HA, Kapur TA, Attawia M, Kraus KH, Holy CE, et al Bone grafts prepared with selective cell retention technology heal canine segmental defects as effectively as autograft. J Orthop Res. 2006;24: 857–866. 10.1002/jor.20094 16602110

[13] Caralla T, Joshi P, Fleury S, Luangphakdy V, Shinohara K, Pan H, et al In vivo transplantation of autogenous marrow-derived cells following rapid intraoperative magnetic separation based on hyaluronan to augment bone regeneration. Tissue Eng Part A. 2013;19: 125–134. 10.1089/ten.tea.2011.0622 23082937 PMC3593694

[14] Giannoni P, Scaglione S, Daga A, Ilengo C, Cilli M, Quarto R. Short-Time Survival and Engraftment of Bone Marrow Stromal Cells in an Ectopic Model of Bone Regeneration. Tissue Eng Part A. 2010;16: 489–499. 10.1089/ten.tea.2009.0041 19712045

[15] Hu X, Yu SP, Fraser JL, Lu Z, Ogle ME, Wang J-A, et al Transplantation of hypoxia-preconditioned mesenchymal stem cells improves infarcted heart function via enhanced survival of implanted cells and angiogenesis. J Thorac Cardiovasc Surg. 2008;135: 799–808. 10.1016/j.jtcvs.2007.07.071 18374759

[16] Zimmermann CE, Gierloff M, Hedderich J, Acil Y, Wiltfang J, Terheyden H. Survival of Transplanted Rat Bone Marrow-Derived Osteogenic Stem Cells In Vivo. Tissue Eng Part A. 2011;17: 1147–1156. 10.1089/ten.tea.2009.0577 21142699

[17] Heylman CM, Caralla TN, Boehm CA, Patterson TE, Muschler GF. Slowing the Onset of Hypoxia Increases Colony Forming Efficiency of Connective Tissue Progenitor Cells In Vitro. J Regen Med Tissue Eng. 2013;2 10.7243/2050-1218-2-7 24371519 PMC3872071

[18] Muschler GF, Nakamoto C, Griffith LG. Engineering principles of clinical cell-based tissue engineering. J Bone Joint Surg Am. 2004;86-A: 1541–1558. 15252108 10.2106/00004623-200407000-00029

[19] Muschler GF, Midura RJ, Nakamoto C. Practical Modeling Concepts for Connective Tissue Stem Cell and Progenitor Compartment Kinetics. Journal of Biomedicine and Biotechnology. Hindawi Publishing Corporation; 2003;2003: 170–193. 10.1155/S1110724303209165 12975533 PMC400211

[20] Tamama K, Fan VH, Griffith LG, Blair HC, Wells A. Epidermal growth factor as a candidate for ex vivo expansion of bone marrow-derived mesenchymal stem cells. Stem Cells. 2006;24: 686–695. 10.1634/stemcells.2005-0176 16150920

[21] Tamama K, Kawasaki H, Wells A. Epidermal Growth Factor (EGF) Treatment on Multipotential Stromal Cells (MSCs). Possible Enhancement of Therapeutic Potential of MSC. Journal of Biomedicine and Biotechnology. Hindawi Publishing Corporation; 2010;2010: 1–10. 10.1182/blood.V97.5.1227 PMC2825653 20182548

[22] Kuznetsov SA, Friedenstein AJ, Robey PG. Factors required for bone marrow stromal fibroblast colony formation in vitro. Br J Haematol. 1997;97: 561–570. 9207401 10.1046/j.1365-2141.1997.902904.x

[23] Gronthos S, Simmons PJ. The growth factor requirements of STRO-1-positive human bone marrow stromal precursors under serum-deprived conditions in vitro. Blood. 1995;85: 929–940. 7849315

[24] Satomura K, Derubeis AR, Fedarko NS, Ibaraki-O'Connor K, Kuznetsov SA, Rowe DW, et al Receptor tyrosine kinase expression in human bone marrow stromal cells. J Cell Physiol. 1998;177: 426–438. 9808151 10.1002/(SICI)1097-4652(199812)177:3<426::AID-JCP6>3.0.CO;2-F

[25] Martineau-Doize B, Lai WH, Warshawsky H, Bergeron JJM. In Vivo Demonstration of Cell Types in Bone That Harbor Epidermal Growth Factor Receptors. Endocrinology. The Endocrine Society; 1988;123: 841–858. 10.1210/endo-123-2-841 3260855

[26] Wang K, Yamamoto H, Chin JR, Werb Z, Vu TH. Epidermal growth factor receptor-deficient mice have delayed primary endochondral ossification because of defective osteoclast recruitment. J Biol Chem. 2004;279: 53848–53856. 10.1074/jbc.M403114200 15456762 PMC2779713

[27] Zhang X, Tamasi J, Lu X, Zhu J, Chen H, Tian X, et al Epidermal growth factor receptor plays an anabolic role in bone metabolism in vivo. J Bone Miner Res. 2011;26: 1022–1034. 10.1002/jbmr.295 21542005 PMC3179301

[28] Wells A, Welsh JB, Lazar CS, Wiley HS, Gill GN, Rosenfeld MG. Ligand-induced transformation by a noninternalizing epidermal growth factor receptor. Science. 1990;247: 962–964. 2305263 10.1126/science.2305263

[29] Countaway JL, Nairn AC, Davis RJ. Mechanism of desensitization of the epidermal growth factor receptor protein-tyrosine kinase. J Biol Chem. 1992;267: 1129–1140. 1309762

[30] Platt MO, Roman AJ, Wells A, Lauffenburger DA, Griffith LG. Sustained epidermal growth factor receptor levels and activation by tethered ligand binding enhances osteogenic differentiation of multi-potent marrow stromal cells. J Cell Physiol. 2009;221: 306–317. 10.1002/jcp.21854 19544388 PMC3084602

[31] Fan VH, Au A, Tamama K, Littrell R, Richardson LB, Wright JW, et al Tethered Epidermal Growth Factor Provides a Survival Advantage to Mesenchymal Stem Cells. Stem Cells. 2007;25: 1241–1251. 10.1634/stemcells.2006-0320 17234993

[32] Wu S, Wells A, Griffith LG, Lauffenburger DA. Controlling multipotent stromal cell migration by integrating “course-graining” materials and ‘fine-tuning’ small molecules via decision tree signal-response modeling. Biomaterials. 2011;32: 7524–7531. 10.1016/j.biomaterials.2011.06.050 21782235 PMC3156355

[33] Rodrigues M, Blair H, Stockdale LN, Griffith L, Wells A. Surface tethered epidermal growth factor protects proliferating and differentiating multipotential stromal cells from FasL-induced apoptosis. Stem Cells. 2013;31: 104–116. 10.1002/stem.1215 22948863 PMC3528829

[34] Platt MO, Wilder CL, Wells A, Griffith LG, Lauffenburger DA. Multipathway Kinase Signatures of Multipotent Stromal Cells Are Predictive for Osteogenic Differentiation. Stem Cells. 2009;27: 2804–2814. 10.1002/stem.215 19750537 PMC2976759

[35] Eniwumide JO, Yuan H, Cartmell SH, Meijer GJ, de Bruijn JD. Ectopic bone formation in bone marrow stem cell seeded calcium phosphate scaffolds as compared to autograft and (cell seeded) allograft. European Cells and Materials. 2007;14: 30–39. 17674330 10.22203/ecm.v014a03

[36] Kai T, Shao-qing G, Geng-ting D. In vivo evaluation of bone marrow stromal-derived osteoblasts-porous calcium phosphate ceramic composites as bone graft substitute for lumbar intervertebral spinal fusion. Spine. 2003;28: 1653–1658. 10.1097/01.BRS.0000083168.37329.B4 12897487

[37] Krebsbach PH, Kuznetsov SA, Satomura K, Emmons R, Rowe DW, Robey PG. Bone formation in vivo: Comparison of osteogenesis by transplanted mouse and human marrow stromal fibroblasts. Transplantation. 1997;63: 1059–1069. 9133465 10.1097/00007890-199704270-00003

[38] Orii H, Sotome S, Chen J, Wang J, Shinomiya K. Beta-tricalcium phosphate (beta-TCP) graft combined with bone marrow stromal cells (MSCs) for posterolateral spine fusion. J Med Dent Sci. 2005 ed. 2005;52: 51–57. 15868741

[39] Damron TA. Use of 3D beta-tricalcium phosphate (Vitoss (R)) scaffolds in repairing bone defects. Nanomedicine. 2007;2: 763–775. 10.2217/17435889.2.6.763 18095844

[40] Bauer TW, Smith ST. Bioactive materials in orthopaedic surgery: overview and regulatory considerations. Clin Orthop Relat Res. 2002;: 11–22. 10.1097/00003086-200202000-00003 11937862

[41] Liu B, Lun D-X. Current application of β-tricalcium phosphate composites in orthopaedics. Orthop Surg. 2012;4: 139–144. 10.1111/j.1757-7861.2012.00189.x 22927147 PMC6583186

[42] Meyers SR, Hamilton PT, Walsh EB, Kenan DJ, Grinstaff MW. Endothelialization of Titanium Surfaces. Adv Mater. 2007;19: 2492–2498. 10.1002/adma.200700029

[43] Whaley SR, English DS, Hu EL, Barbara PF, Belcher AM. Selection of peptides with semiconductor binding specificity for directed nanocrystal assembly. Nature. 2000;405: 665–668. 10.1038/35015043 10864319

[44] Sanghvi AB, Miller KPH, Belcher AM, Schmidt CE. Biomaterials functionalization using a novel peptide that selectively binds to a conducting polymer. Nat Mater. 2005;4: 496–502. 10.1038/nmat1397 15895095

[45] Sano K-I, Sasaki H, Shiba K. Specificity and biomineralization activities of Ti-binding peptide-1 (TBP-1). Langmuir. 2005;21: 3090–3095. 10.1021/la047428m 15779989

[46] Saini S, Muschler G, McGlohorn J. The effect of bone graft architecture on bone formation in a canine defect model Orthopaedic Research Society, Chicago, IL 2006.

[47] Luangphakdy V, Walker E, Shinohara K, Pan H, Hefferan T, Bauer TW, et al Evaluation of Osteoconductive Scaffolds in the Canine Femoral Multi-Defect Model. Tissue Eng Part A. 2013;19: 634–648. 10.1089/ten.tea.2012.0289 23215980 PMC3568967

[48] Jay SM, Kurtagic E, Alvarez LM, de Picciotto S, Sanchez E, Hawkins JF, et al Engineered Bivalent Ligands to Bias ErbB Receptor-mediated Signaling and Phenotypes. J Biol Chem. 2011;286: 27729–27740. 10.1074/jbc.M111.221093 21622572 PMC3149363

[49] Roh JD, Nelson GN, Udelsman BV, Brennan MP, Lockhart B, Fong PM, et al Centrifugal seeding increases seeding efficiency and cellular distribution of bone marrow stromal cells in porous biodegradable scaffolds. Tissue Engineering. 2007;13: 2743–2749. 10.1089/ten.2007.0171 17880269

[50] Kuhl PR, Griffith-Cima LG. Tethered epidermal growth factor as a paradigm for growth factor-induced stimulation from the solid phase. Nature Medicine. 1996;2: 1022–1027. 8782461 10.1038/nm0996-1022

[51] Marcantonio NA, Boehm CA, Rozic RJ, Au A, Wells A, Muschler GF, et al The influence of tethered epidermal growth factor on connective tissue progenitor colony formation. Biomaterials. 2009;30: 4629–4638. 10.1016/j.biomaterials.2009.05.061 19540579 PMC3119364

[52] Ito Y, Li JS, Takahashi T, Imanishi Y, Okabayashi Y, Kido Y, et al Enhancement of the mitogenic effect by artificial juxtacrine stimulation using immobilized EGF. J Biochem. 1997;121: 514–520. 9133620 10.1093/oxfordjournals.jbchem.a021616

[53] Ito Y, Chen G, Imanishi Y, Morooka T, Nishida E, Okabayashi Y, et al Differential control of cellular gene expression by diffusible and non-diffusible EGF. J Biochem. 2001;129: 733–737. 11328595 10.1093/oxfordjournals.jbchem.a002913

[54] Achille V, Mantelli M, Arrigo G, Novara F, Avanzini MA, Bernardo ME, et al Cell-cycle phases and genetic profile of bone marrow-derived mesenchymal stromal cells expanded in vitro from healthy donors. J Cell Biochem. 2011;112: 1817–1821. 10.1002/jcb.23100 21400572

[55] Brounts SH, Lee JS, Weinberg S, Lan Levengood SK, Smith EL, Murphy WL. High Affinity Binding of an Engineered, Modular Peptide to Bone Tissue. Mol Pharmaceutics. American Chemical Society; 2013;10: 2086–2090. 10.1021/mp300662r 23506396 PMC3670755

[56] Lee JS, Wagoner Johnson AJ, Murphy WL. A Modular, Hydroxyapatite-Binding Version of Vascular Endothelial Growth Factor. Adv Mater. 2010;22: 5494–5498. 10.1002/adma.201002970 20941802

[57] Kang J, Tada S, Sakuragi M, Abe H, Ito R, Ishikawa J, et al An epidermal growth factor derivative with binding affinity for hydroxyapatite and titanium surfaces. Biomaterials. 2013;34: 9747–9753. 10.1016/j.biomaterials.2013.09.004 24055522

[58] Muschler GF, Midura RJ. Connective tissue progenitors: practical concepts for clinical applications. Clin Orthop Relat Res. 2002nd ed. 2002;: 66–80. 10.1097/00003086-200202000-00008 11937867

[59] Lopes MA, Monteiro FJ, Santos JD, Serro AP, Saramago B. Hydrophobicity, surface tension, and zeta potential measurements of glass-reinforced hydroxyapatite composites. J Biomed Mater Res. 1999;45: 370–375. 10321710 10.1002/(sici)1097-4636(19990615)45:4<370::aid-jbm12>3.0.co;2-0

[60] Santana H, González Y, Campana PT, Noda J, Amarantes O, Itri R, et al Screening for stability and compatibility conditions of recombinant human epidermal growth factor for parenteral formulation: Effect of pH, buffers, and excipients. International Journal of Pharmaceutics. 2013;452: 52–62. 10.1016/j.ijpharm.2013.04.054 23624083

[61] Kitajima T, Sakuragi M, Hasuda H, Ozu T, Ito Y. A chimeric epidermal growth factor with fibrin affinity promotes repair of injured keratinocyte sheets. Acta Biomaterialia. 2009;5: 2623–2632. 10.1016/j.actbio.2009.03.022 19376761

[62] Dorozhkin SV. Biphasic, triphasic and multiphasic calcium orthophosphates. Acta Biomaterialia. 2012;8: 963–977. 10.1016/j.actbio.2011.09.003 21945826

[63] Nam KT. Virus-Enabled Synthesis and Assembly of Nanowires for Lithium Ion Battery Electrodes. Science. 2006;312: 885–888. 10.1126/science.1122716 16601154

[64] Roy MD, Stanley SK, Amis EJ, Becker ML. Identification of a Highly Specific Hydroxyapatite-binding Peptide using Phage Display. Adv Mater. 2008;20: 1830–1836. 10.1002/adma.200702322

[65] Naik RR, Stringer SJ, Agarwal G, Jones SE, Stone MO. Biomimetic synthesis and patterning of silver nanoparticles. Nat Mater. 2002;1: 169–172. 10.1038/nmat758 12618805

[66] Sano K-I, Shiba K. A Hexapeptide Motif that Electrostatically Binds to the Surface of Titanium. J Am Chem Soc. 2003;125: 14234–14235. 10.1021/ja038414q 14624545

[67] Kriplani U, Kay BK. Selecting peptides for use in nanoscale materials using phage-displayed combinatorial peptide libraries. Current Opinion in Biotechnology. 2005;16: 470–475. 10.1016/j.copbio.2005.07.001 16019201

[68] Seker UOS, Wilson B, Sahin D, Tamerler C, Sarikaya M. Quantitative Affinity of Genetically Engineered Repeating Polypeptides to Inorganic Surfaces. Biomacromolecules. 2009;10: 250–257. 10.1021/bm8009895 19072301

[69] Wang S, Humphreys ES, Chung S-Y, Delduco DF, Lustig SR, Wang H, et al Peptides with selective affinity for carbon nanotubes. Nat Mater. 2003;2: 196–200. 10.1038/nmat833 12612679

[70] NEB. Ph.D. Phage Display Library Instruction Manual. In: Ph.D. Phage Display Library Instruction Manual [Internet]. [cited 7 Sep 2014]. Available: http://www.neb.com/~/media/Catalog/All-Products/BDA9A6DB00DC42E8B93A8D8FBD08C49B/Datacards%20or%20Manuals/manualE8100.pdf.

[71] Malik P, Perham RN. New vectors for peptide display on the surface of filamentous bacteriophage. Gene. 1996;171: 49–51. 10.1016/0378-1119(96)00070-4 8675029

[72] Goldsmith ME, Konigsberg WH. Adsorption protein of the bacteriophage fd: isolation, molecular properties, and location in the virus. Biochemistry. American Chemical Society; 1977;16: 2686–2694. 10.1021/bi00631a016 329863

[73] Woolford JL, Steinman HM, Webster RE. Adsorption protein of bacteriophage fl: solubilization in deoxycholate and localization in the fl virion. Biochemistry. 1977;16: 2694–2700. 10.1021/bi00631a017 329864

[74] Lin TC, Webster RE, Konigsberg W. Isolation and characterization of the C and D proteins coded by gene IX and gene VI in the filamentous bacteriophage fl and fd. J Biol Chem. 1980;255: 10331–10337. 7000776

[75] Smith GP. Surface presentation of protein epitopes using bacteriophage expression systems. Current Opinion in Biotechnology. 1991;2: 668–673. 10.1016/0958-1669(91)90032-Z 1370065

[76] Weiger MC, Park JJ, Roy MD, Stafford CM, Karim A. Quantification of the binding affinity of a specific hydroxyapatite binding peptide. Biomaterials. 2010. 10.1016/j.biomaterials.2010.01.012 20106520

[77] Seker UOS, Wilson B, Dincer S, Kim IW, Oren EE, Evans JS, et al Adsorption behavior of linear and cyclic genetically engineered platinum binding peptides. Langmuir. 2007;23: 7895–7900. 10.1021/la700446g 17579466

[78] Seker UOS, Demir HV. Material Binding Peptides for Nanotechnology. Molecules. 2011;16: 1426–1451. 10.3390/molecules16021426 21307821 PMC6259601

[79] Clackson T, Lowman HB. Phage Display: A Practical Approach—Google Books. 2004.

[80] Scott J, Smith G. Searching for peptide ligands with an epitope library. Science. 1990;249: 386–390. 10.1126/science.1696028 1696028

[81] Urresti S, Albesa-Jové D, Schaeffer F, Pham HT, Kaur D, Gest P, et al Mechanistic insights into the retaining glucosyl-3-phosphoglycerate synthase from mycobacteria. J Biol Chem. 2012;287: 24649–24661. 10.1074/jbc.M112.368191 22637481 PMC3397893

[82] Webster MPJ, Jukes R, Zamfir VS, Kay CWM, Bagnéris C, Barrett T. Crystal structure of the UvrB dimer: insights into the nature and functioning of the UvrAB damage engagement and UvrB-DNA complexes. Nucleic Acids Res. 2012;40: 8743–8758. 10.1093/nar/gks633 22753105 PMC3458569

[83] Obmolova G, Teplyakov A, Bonander N, Eisenstein E, Howard AJ, Gilliland GL. Crystal structure of dephospho-coenzyme A kinase from Haemophilus influenzae. J Struct Biol. 2001;136: 119–125. 10.1006/jsbi.2001.4428 11886213

[84] Kozlowski H, Swiatek J, Siatecki Z. 1H NMR studies on interactions of calcium and magnesium with aspartic acid and asparagine. Acta Biochim Pol. 1981;28: 1–9. 7282211

[85] Itoh D, Yoneda S, Kuroda S, Kondo H, Umezawa A, Ohya K, et al Enhancement of osteogenesis on hydroxyapatite surface coated with synthetic peptide (EEEEEEEPRGDT)in vitro. J Biomed Mater Res. 2002;62: 292–298. 10.1002/jbm.10338 12209950

[86] Fujisawa R, Wada Y, Nodasaka Y, Kuboki Y. Acidic amino acid-rich sequences as binding sites of osteonectin to hydroxyapatite crystals. Biochimica et Biophysica Acta (BBA)—Protein Structure and Molecular Enzymology. 1996;1292: 53–60. 10.1016/0167-4838(95)00190-5 8547349

[87] Fujisawa R, Mizuno M, Nodasaka Y, Yoshinori K. Attachment of osteoblastic cells to hydroxyapatite crystals by a synthetic peptide (Glu7-Pro-Arg-Gly-Asp-Thr) containing two functional sequences of bone sialoprotein. Matrix Biology. 1997;16: 21–28. 10.1016/S0945-053X(97)90113-X 9181551

[88] Kasugai S, Fujisawa R, Waki Y, Miyamoto K-I, Ohya K. Selective Drug Delivery System to Bone: Small Peptide (Asp)6 Conjugation. J Bone Miner Res. 2010;15: 936–943. 10.1359/jbmr.2000.15.5.936 10804024

[89] Sawyer AA, Weeks DM, Kelpke SS, McCracken MS, Bellis SL. The effect of the addition of a polyglutamate motif to RGD on peptide tethering to hydroxyapatite and the promotion of mesenchymal stem cell adhesion. Biomaterials. 2005;26: 7046–7056. 10.1016/j.biomaterials.2005.05.006 15964067

[90] Hunter GK, Goldberg HA. Modulation of crystal formation by bone phosphoproteins: role of glutamic acid-rich sequences in the nucleation of hydroxyapatite by bone sialoprotein. Biochemical Journal. Portland Press Ltd; 1994;302: 175 7915111 10.1042/bj3020175 PMC1137206

[91] Hunter GK, Kyle CL, Goldberg HA. Modulation of crystal formation by bone phosphoproteins: structural specificity of the osteopontin-mediated inhibition of hydroxyapatite formation. Biochemical Journal. Portland Press Ltd; 1994;300: 723 8010953 10.1042/bj3000723 PMC1138226

[92] Hunter GK, Hauschka PV, Poole AR, Rosenberg LC, Goldberg HA. Nucleation and inhibition of hydroxyapatite formation by mineralized tissue proteins. biochemjorg. Portland Press Ltd; 1996. 10.1042/bj3170059 PMC1217486 8694787

[93] Carmona P, Rodriguez ML. Hydrogen bonds between protein side chains and phosphates and their role in biological calcification. Biophysical Chemistry. 1987;28: 161–167. 10.1016/0301-4622(87)80085-6 2447975

[94] Arap MA. Phage display technology: applications and innovations. Genet Mol Biol. 2005;28: 1–9. 10.1590/S1415-47572005000100001

[95] Vendruscolo M, Paci E, Dobson CM, Karplus M. Three key residues form a critical contact network in a protein folding transition state. Nature. 2001;409: 641–645. 10.1038/35054591 11214326

