# Supplementary material for: Tethering of Epidermal Growth Factor (EGF) to Beta Tricalcium Phosphate (βTCP) via Fusion to a High Affinity, Multimeric βTCP-Binding Peptide: Effects on Human Multipotent Stromal Cells/Connective Tissue Progenitors
Source: PLoS One. 2015 Jun 29;10(6):e0129600. doi: 10.1371/journal.pone.0129600 (PMC4488278; doi:10.1371/journal.pone.0129600)
Supplement: S1 Table — (DOCX) [file pone.0129600.s001.docx]

| Name | Sequence |
| --- | --- |
| MBP | MKIEEGKLVIWINGDKGYNGLAEVGKKFEKDTGIKVTVEHPDKLEEKFPQVAATGDGPDIIFWAHDRFGGYAQSGLLAEITPDKAFQDKLYPFTWDAVRYNGKLIAYPIAVEALSLIYNKDLLPNPPKTWEEIPALDKELKAKGKSALMFNLQEPYFTWPLIAADGGYAFKYENGKYDIKDVGVDNAGAKAGLTFLVDLIKNKHMNADTDYSIAEAAFNKGETAMTINGPWAWSNIDTSKVNYGVTVLPTFKGQPSKPFVGVLSAGINAASPNKELAKEFLENYLLTDEGLEAVNKDKPLGAVALKSYEEELVKDPRIAATMENAQKGEIMPNIPQMSAFWYAVRTAVINAASGRQTVDEALKDAQTNSSSSSNNNNNNNNNNIEGRGS |
| BP_10_ | LLADTTHHRPWTLLADTTHHRPWTLLADTTHHRPWTLLADTTHHRPWTLLADTTHHRPWTLLADTTHHRPWTLLADTTHHRPWTLLADTTHHRPWTLLADTTHHRPWTLLADTTHHRPWT |
| Tether (-T-) | ASGAGGSEGGGSEGGTSGATGAGTSTSGGGASTGGGLEIRAAFLRRRNTALRTRVAELRQRVQRLRNIVSQYETRYGPLTGASGAGGSEGGGSEGGTSGATGAGTS |
| hEGF | NSDSECPLSHDGYCLHDGVCMYIEALDKYACNCVVGYIGERCQYRDLKWWELR |

**Table S1. BP_10_-T-EGF full protein sequence**
